# The Optimal Protective 25-Hydroxyvitamin D Level for Different Health Outcomes in Adults: A Brief Summary of Dose–Response Meta-Analyses

**DOI:** 10.3390/metabo15040264

**Published:** 2025-04-10

**Authors:** Cem Ekmekcioglu, Michael Poteser

**Affiliations:** Department of Environmental Health, Center for Public Health, Medical University of Vienna, 1090 Vienna, Austria; michael.poteser@meduniwien.ac.at

**Keywords:** vitamin D, 25-hydroxyvitamin D, risk reduction, disease, optimal level

## Abstract

Vitamin D is very important for bone metabolism as well as for the prevention of various diseases, such as type 2 diabetes, cardiovascular disease and different types of cancer. Although vitamin D deficiency is widespread and an important public health problem, there exists controversy in the scientific community, with no established standard definition of adequate and deficient vitamin D status. To add new information on this topic, the aim of this brief opinion paper is to identify and discuss the optimal 25(OH)D concentration (range) for a reduction in the risk of various disease outcomes by summarizing dose–response reporting meta-analyses.

## 1. Introduction

The best method to determine the vitamin D status and, in particular, a vitamin D deficiency, is the measurement of serum 25-hydroxyvitamin D (25(OH)D), which reflects both the dietary vitamin D intake and sunlight exposure [1]. Serum 25(OH)D levels are regarded as optimal when the blood level is sufficient to maximally suppress serum parathyroid hormone (PTH). However, former studies showed a huge variation in maximal PTH suppression at levels between 20/25 and 110/125 nmol/L of serum 25(OH)D (summarized in [2,3]), and they found that PTH levels might begin to plateau at >65 nmol/L [4]. More recent studies have shown an inverse correlation between PTH and 25(OH)D levels for the whole range of 25(OH)D concentrations, without reaching a plateau [5,6].

There has been a controversy about what exact 25(OH)D concentrations define vitamin D deficiency and sufficiency. The Institute of Medicine (IOM, U.S. National Academy of Sciences) considers the minimal 25(OH)D concentration of 20 ng/mL (50 nmol/L) as physiologically adequate for at least 97.5% of the population [7]. The Endocrine Society, in 2011, recommended serum levels of >30 ng/mL (>75 nmol/L) as optimal [8], although in their revised 2024 statement they did not provide reference values for optimal serum 25(OH)D concentrations and stated that “in healthy adults, 25(OH)D levels that provide outcome-specific benefits have not been established in clinical trials” [9].

Vitamin D is primarily linked to calcium and phosphorus metabolism and bone health. However, especially in the last two decades, observational studies have also shown an inverse association between the vitamin D status and the risk of various diseases such as cancer, diabetes or cardiovascular and certain autoimmune diseases [1,10].

In a narrative review, it was suggested that for different health outcomes, like the bone mineral density, lower extremity function and fall or fracture prevention, a serum 25(OH)D level around 75 nmol/L should be the target [11], although possible optimal levels for other outcomes like cancer prevention appear to be higher, in the range of 75–110 nmol/L [11].

Also, others have suggested optimal serum 25(OH)D levels for a reduction in the incidence of breast and colorectal cancer to be higher than 100 nmol/L [12].

In another review, the optimal 25(OH)D concentration for various outcomes, like all-cause mortality, cancer, type 2 diabetes or cardiovascular disease, were summarized as lying between 25 ng/mL (62.5 nmol/L) and 60 ng/mL (150 nmol/L) [10].

At a 2-day Vitamin D Summit Meeting of 25 experts held on 7–8 November 2009 in Paris, it was concluded that the 25(OH)D level in specific groups of patients with or at risk of problems of the musculoskeletal system, cardiovascular diseases, autoimmune diseases and cancer should be above 30 ng/mL (75 nmol/L) for optimal health benefits [13].

Furthermore, to achieve the pleiotropic, non-skeletal effects of vitamin D, a recommendation of 30–50 ng/mL (75–125 nmol/L) was provided in a multi-expert publication from 2018 [14].

Finally, in a workshop report and review from the Netherlands, age-dependent values were proposed with 50–75 nmol/L possibly being the optimal range for an age range of 5–64 years and 75–100 nmol/L for those older than 65 years to ensure an optimal anti-fracture effect [15].

All in all, in general, the majority of disease-specific recommendations to date have set a lower limit of 75 nmol/L and an upper one of about 125 nmol/L for optimal 25(OH)D levels.

To add new information on the topic of optimal 25(OH)D levels, the aim of this brief summary is to identify and discuss the 25(OH)D concentration (range) for optimal risk reduction for various disease outcomes by, probably for the first time, summarizing and evaluating data from meta-analyses providing dose–response curves to identify the concentration-dependent lowest risk levels.

## 2. Methods

A search was conducted on 5 February 2024 in PubMed with the following search terms: “dose response” AND (“vitamin d status” OR “25OHD” OR “25 hydroxyvitamin D” OR “calcitriol”) AND (“meta-analysis” OR “systematic review”).

Meta-analyses were only included if they provided a dose–response curve with values of the relative risk (RR), an odds ratio (OR) or a hazard ratio (HR) as a function of the 25(OH)D levels for different disease outcomes. (Approximate) data of the lowest RR/OR/HR were taken from the publications, and, if not presented, estimated through visual inspection from the dose–response curves. In unclear cases, computerized curve analysis (Engauge Digitizer Software, https://sourceforge.net/projects/digitizer/, accessed on 6 April 2025) was used to confirm the visual estimation. In the case of linear or almost linear associations, the endpoint of the curve/line was taken as the lowest risk value.

## 3. Results

**Figure 1:**
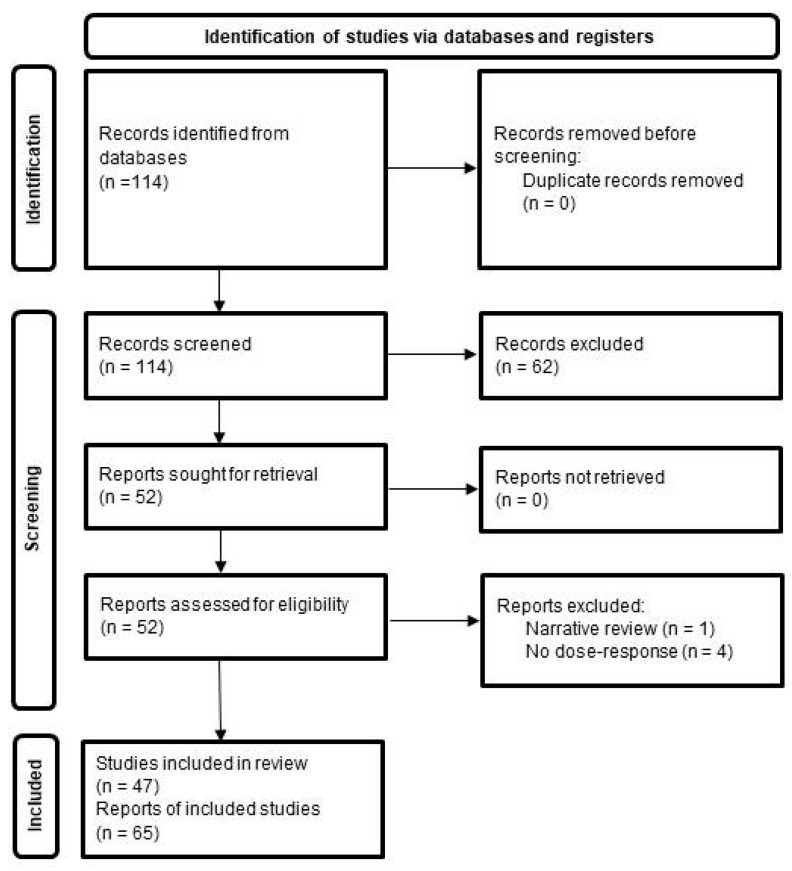
Flow diagram of the search strategy and study selection. Source: [16]. This work is licensed under CC BY 4.0. To view a copy of this license, visit https://creativecommons.org/licenses/by/4.0/, accessed on 6 April 2025.

The search yielded 113 papers, from which 51 were extracted after checking the titles and abstracts. From these, five were excluded since one was a narrative review, two did not provide dose–response curves related to the 25(OH)D status and the remaining two did not provide dose–response curves at all. In addition to the PubMed search, one study was additionally found through an individual search. So, a total of 47 papers with 65 analyzed outcomes were included in this summary (Figure 1, Table 1).

In addition to the all-cause and disease-specific mortality, various other outcomes including, in particular, different types of cancer and also metabolic or cardiovascular diseases like diabetes or stroke, were addressed in the reviewed papers (Table 1).

The lowest risk for most of the different outcomes was found at 25(OH)D levels between approximately 40–50 nmol/L and 100 nmol/L (Table 1), with about half of the analyzed outcomes showing the lowest risk at ≤75 nmol/L. Only a few had a lowest risk estimation higher than 100 nmol/L.

**Figure 2:**
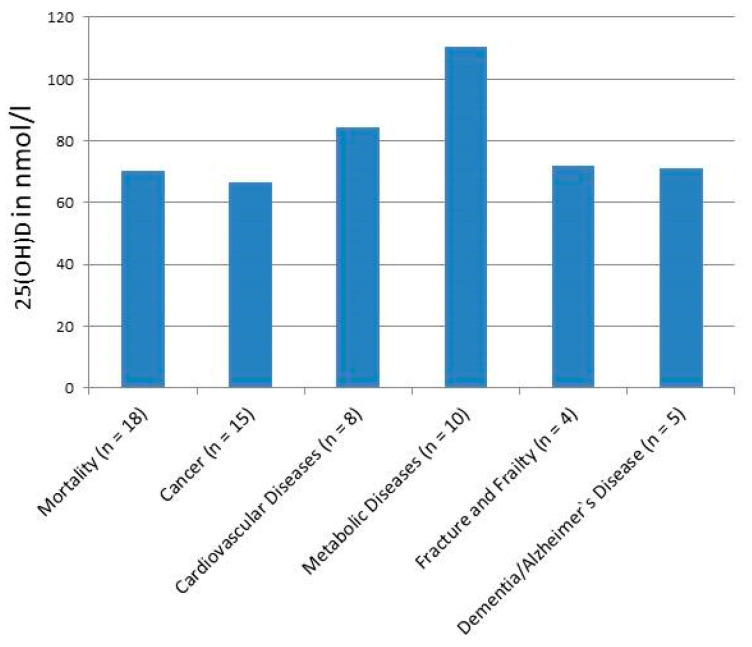
Lowest risk (RR/HR/OR) of 25-hydroxyvitamin D concentration (in nmol/L) in dose–response meta-analyses for different outcome groups. Data are taken from Table 1 and the mean values of the lowest risk concentrations from the respective studies are presented.

Grouping individual studies into different outcome groups (with ≥3 studies) showed that for most of the combined outcomes, the mean 25(OH)D values were between approximately 60 and 80 nmol/L (Figure 2) with the exception of metabolic diseases, which included diabetes, metabolic syndrome, obesity and dyslipidemia, showing a combined mean value of 111 nmol/L.

Our survey further showed that the dose–response curve for 25(OH)D and various outcomes only showed a clear optimal concentration, in the sense that 25(OH)D levels above the optimum may increase the risks, for about 40% of the included meta-analyses. Several outcomes were shown to be associated in a linear manner, while other risk endpoints were negatively related to 25(OH)D blood levels and showed a flattening of the curve at higher levels, indicating an asymptotic trend.

## 4. Discussion

Vitamin D deficiency is common in different populations worldwide [63]. 25(OH)D is the most abundant vitamin D metabolite in the circulation and, due to a long half-life of 2–3 weeks, is considered the best indicator of the vitamin D status [1]. Sufficient, repeated evidence is available that the serum 25(OH)D levels are associated with mortality and different clinical outcomes involving major organ systems [64]. However, the optimal target concentration for 25(OH)D still differs between various organizations. Although there is a consensus that very low levels of less than 25–30 nmol/L indicate a clinically relevant (severe) deficiency, primarily because of an increased risk for rickets/osteomalacia, the establishment of higher thresholds is still under discussion, with some organizations like the IOM setting 50 nmol/L as adequate for most of the population [7], while others recommend higher optimal levels, also dependent on different periods of life and clinical conditions (reviewed in [65]). For example, in a recent publication by a large group of experts, adequate to optimal 25(OH)D levels (for Poland) were indicated as being 75–125 nmol/L [66]. By setting a realistic upper level of 125 nmol/L for adequate 25(OH)D levels, this expert group also considered the important risk of vitamin D intoxication beyond concentrations of 250 nmol/L [66].

By inspecting a large number of dose–response curves from meta-analyses, we found that in nearly half of the studies, the lowest risks were found to be associated with levels lower than 75 nmol/L, and when looking at different outcome groups, there seemed to be a trend for higher optimal 25(OH)D concentrations in metabolic diseases. For example, in a systematic review of three vitamin D supplementation trials, which investigated the risk of new-onset diabetes in adults with prediabetes, it was found that in participants with 25(OH)D levels of 100 to 124 nmol/L and 125 nmol/L or higher during follow-up, the hazard ratios for diabetes were 0.38 (CI: 0.27 to 0.55) and 0.24 (CI: 0.16 to 0.36), respectively, compared with participants who maintained levels of 50 to 74 nmol/L [67].

Through multiple mechanisms, like inducing genes related to glucose transport or affecting intracellular calcium levels in β-cells, vitamin D is involved in the function and secretion of insulin [68]. Higher vitamin D levels therefore might be advantageous in decreasing the diabetes risk in a dose-dependent manner.

In contrast to metabolic diseases, the concentrations associated with the mean lowest risks were lower for mortality (all-cause and disease-specific), cancer and cardiovascular diseases. Regarding cardiovascular diseases, a previous study, for example, showed a U-shaped association, with the lowest risk for acute coronary syndrome and all mortality lying between 50 and 90 nmol/L 25(OH)D and lower and higher levels being associated with an increased risk [69]. An increased risk for major cardiac and cerebrovascular events at 25(OH)D levels > 100 nmol/L compared to those of 75–100 nmol/L was also calculated in cardiac surgical patients [70]. Also, regarding lung cancer, for example, a U-shaped association with the lowest risk values at a 25(OH)D concentration between approximately 50 until 90 nmol/L was found [28].

The relevance of potential different protective optimal 25(OH)D levels for various outcome groups might be, for example, in certain cases, like in the case of a high risk for diabetes or metabolic syndrome, where the supplemented dose of vitamin D can be increased to reach the desired levels in the direction of 100 nmol/L 25(OH)D. However, this approach could also be a double-edged sword with beneficial effects for one outcome and suboptimal effects for another. More studies and specific knowledge are necessary to handle this in an evidence-based and cautious manner.

A major limitation of this brief opinion review is that, due to the objective of this opinion paper, only meta-analyses providing dose–response curves were included. Therefore, other important vitamin D-related clinical outcomes, for which, in our search, no dose–response meta-analyses were found could not be assessed. One of these was infections, and especially COVID-19, with several meta-analyses suggesting that a low vitamin D status is associated with an increased infection risk or severe outcomes (reviewed in [71]).

In conclusion, to define a universal level that could be considered optimal for minimizing overall risks, considering the linear and nonlinear outcome relationships of multiple endpoints, is rather a challenge, and it is not possible to determine a common optimal concentration that minimizes the risk for all of these outcomes. All in all, the optimal vitamin D status seems to be tissue-dependent and might also vary by age and race, which would make it difficult to set generally applicable optimal values.

Nevertheless, there seems to be little evidence that 25(OH)D concentrations higher than 100 nmol/L provide further risk reduction, which could be due to the limited number of participants with very high 25(OH)D levels in the studies.

Well-designed and -monitored intervention trials of treatment for various clinical outcomes and 25(OH)D targets might reveal more information about the optimal protective vitamin D status.

## Figures and Tables

**Table 1:** 25-hydroxyvitamin D levels in dose–response meta-analyses of different outcomes.

Reference	Outcome(s)	Included Studies for Dose–Response Analysis	Lowest Risk (RR/HR/OR) in nmol/L *	Shape of Association—Nonlinearity (Significance)
Gorham ED et al. 2007 [17]	Colorectal cancer	5 studies	34 ng/mL (85 nmol/L, 50% reduction in incidence, from paper)	Inverse dose–response gradient in quintiles
Grant WB 2010 [12]	Breast cancer	6 studies	Approx. 78 nmol/L (50% reduction in incidence rate, from paper)	Nonlinear regression line
	Colorectal cancer	10 studies	Approx. 60 nmol/L (50% reduction in incidence rate, from paper)	
Chung M et al. 2011 [18]	Colorectal cancer	9 studies	Lowest risk not clearly extractable; especially for colorectal cancer, most studies found inverse relationship with prediagnosis blood 25- (OH)D concentration	Presentation of individual study curves
	Prostate cancer	8 studies		
	Breast cancer	4 studies		
Bischoff-Ferrari HA et al. 2012 [19]	Hip fracture	4383 study participants	≥61 nmol/L (from paper)	Threshold assessment for risk of fracture according to quartile of baseline 25(OH)D level
	Non-vertebral fracture		≥61 nmol/L (from paper)	
Wang L et al. 2012 [20]	Cardiovascular disease	16 studies	Approx. 60 nmol/L (from paper)	Linear relation (*p* = 0.06); higher risk below 50–60 nmol/L; high values not clearly associated with higher risk
Song Y et al. 2013 [21]	Type 2 diabetes mellitus	18 studies	Significantly lower risk at approximately 50 nmol/L (from paper)	Linear relation across range of 25(OH)D concentration from 20 up to 160 nmol/L was significant (*p* < 0.0001)
			Around 100 nmol/L (after exclusion of 3 studies; evidence for relation of 25(OH)D concentration of >100 nmol/L with type 2 diabetes was weak)	
Bauer SR et al. 2013 [22]	Breast cancer in postmenopausal women	Total of 9 studies	35 ng/mL (87.5 nmol/L, from paper)	Nonlinear association (*p* = 0.05)
Schöttker B et al. 2013 [23]	Overall mortality	12 studies	Approx. 50–60 nmol/L in most of the studies (rather linear, weak association)	25(OH)D concentration categories in single studies
Ju SY et al. 2014 [24]	Metabolic syndrome	16 cross-sectional studies	120 nmol/L (from paper; possibly lower risk at higher levels according to regression model)	Weighted linear regression model was fitted (*p* for linear trend < 0.001)
Maalmi H et al. 2014 [25]	Overall mortality, breast cancer	5 studies	Approx. 50–100 nmol/L (estimate)	25(OH)D concentration categories in single studies
	Overall mortality, colorectal cancer	5 studies	Approx. 50–100 nmol/L (estimate) Few data points, somewhat high deviation	
Schöttker B et al. 2014 [26]	All-cause mortality	8 studies	70 nmol/L	Curvilinear association within quintiles of 25(OH)D concentration
	Cardiovascular mortality (with or without history of CVD)	8 studies	70 nmol/L	
Garland CF et al. 2014 [27]	All-cause mortality	32 studies	30–39 ng/mL (75–97.5 nmol/L), with 36 ng/mL (90 nmol/L) n.s. at higher levels (from paper)	Stratified in 10 ng/mL intervals
Chen GC et al. 2015 [28]	Lung cancer	10 studies (?)	Approximately 53 nmol/L (from paper)	U-shaped, nonlinear relationship (*P*_nonlinearity_ = 0.02)
Mohr SB et al. 2015 [29]	Colorectal cancer mortality	4 studies	Approx. 30–40 ng/mL (75–100 nmol/L)	Results of individual studies
Zhao Y et al. 2016 [30]	Bladder cancer	7 studies	75 nmol/L (last quintile)	Inverse linear in quintiles
Ekmekcioglu C et al. 2017 [31]	Type 2 diabetes	119 risk estimates	About 65 ng/mL (162.5 nmol/L, from paper)	Roughly U-shaped association
	Colorectal cancer	111 risk estimates	About 55 ng/mL (137.5 nmol/L, from paper)	U-shaped association
Feng Q et al. 2017 [32]	Lung cancer	9 studies	Around 43 nmol/L (estimate)	Roughly U-shaped
Zhang R et al. 2017 [33]	Total cardiovascular events	32 publications	Approx. 25 ng/mL (62.5 nmol/L, from paper)	Nonlinear association (*p* < 0.001)
	CVD mortality	17 publications	40 ng/mL (100 nmol/L, end of curve, estimate)	Nonlinear association (*p* < 0.022)
LV QB et al. 2017 [34]	Hip fracture	4 studies	Approx. 60 nmol/L (from paper)	*p* = 0.110 for nonlinearity
Jayedi A et al. 2017 [35]	All-cause mortality in patients with chronic kidney disease	6–7 studies	Approx. 25–30 ng/mL (62.5–75 nmol/L, from paper)	Nonlinear dose–response meta-analysis, significant curvilinear association (*P*_nonlinearity_ = 0.002 and 0.004 after exclusion of one study)
Garland CF, Gorham ED 2017 [36]	Risk of colorectal cancer	15 studies	Suggested to be 35 ng/mL (87.5 nmol/L, from paper)	Linear downward trend, medians of ORs for each 10 ng/mL interval
Maalmi H et al. 2018 [37]	Overall survival in colorectal cancer patients	4 studies	Around 40–50 nmol/L (rough estimate)	25(OH)D concentration categories in single studies
	Cancer-specific survival	3 studies	Around 40–50 nmol/L (rough estimate)	25(OH)D concentration categories in single studies
Wei H et al. 2018 [38]	Lung cancer	9 studies	Around 60 nmol/L (estimate)	Nonlinear model, nonlinearity tests (*p* = 0.14)
Hu K et al. 2018 [39]	Overall survival in breast cancer patients	6 studies	Linear decrease (unreliable data in the highest range)	No significant nonlinearity in relationship between overall survival and circulating 25(OH)D levels (*P*_nonlinearity_ = 0.13)
Ju SY et al. 2018 [40]	Frailty syndrome	4 cohort studies, 6 cross-sectional studies	94 nmol/L (lowest RR, from paper)	Linear model
Chen H et al. 2018 [41]	Dementia	9 studies	Approx. 65 nmol/L (end of linear trend, estimate)	Inverse linear trend (*p* < 0.001), nonlinearity n.s.
	Alzheimer’s disease	4 studies	Approx. 65 nmol/L (end of linear trend)	
Han J et al. 2019 [42]	Total cancer incidence	Not indicated for dose–response analyses	Around 30–50 nmol/L (estimation from curve)	Dose–response linear trend (variance-weighted least squares regression of fixed effect model)
	Total cancer mortality	Not indicated for dose–response analyses	Around 75 nmol/L (estimation from curve)	Dose–response linear trend (variance-weighted least squares regression of fixed effect model)
Zhang L et al. 2019 [43]	Colorectal cancer	4 studies	Around 37 ng/mL (92.5 nmol/L, end of curve, estimation)	Linear and spline model, nonlinear trend (*P*_nonlinearity_ = 0.11)
Yang J et al. 2019 [44]	Mortality of cardiovascular disease		Approx. 90 nmol/L (end of curve, almost linear, estimate)	Nonlinear dose relationship, *p* < 0.001
Li H et al. 2019 [45]	Depression	6 studies	Approx. 65 ng/mL (162.5 nmol/L, end of line, estimate)	Restricted cubic splines, linear association (*P*_nonlinearity_ = 0.96)
Jayedi A et al. 2019 [46]	Dementia	6 studies	25 ng/mL (62.5 nmol/L, from paper)	*P*_nonlinearity_ = 0.05, U-shaped
		5 studies	Approx. 30 ng/mL (75 nmol/L, after exclusion of one study, from paper)	*P*_nonlinearity_ = 0.22
	Alzheimer’s disease	4 studies	35 ng/mL (87.5 nmol/L, from paper)	*P*_nonlinearity_ = 0.08
Shi H et al. 2020 [47]	Stroke	8 cohort studies	50 nmol/L (from paper)	Nonlinear association (*p* = 0.04)
Mahamat-Saleh Y et al. 2020 [48]	Melanoma	3 cohort studies	Around 30 nmol/L (estimate)	Nonlinearity n.s. (*P*_nonlinearity_ = 0.08)
	Keratinocyte cancer	3 cohort studies	Less or more than 60 nmol/L (from paper)	Nonlinear association (*P*_nonlinearity_ = 0.01); inverse U-shaped, highest risk around 60 nmol/L (from paper)
Wu G et al. 2020 [49]	All-cause mortality	Total of 17 studies (dose–response not indicated)	Approx. 40 nmol/L (estimate)	L-shaped
	Colorectal cancer mortality		Approx. 80 nmol/L (estimate)	Nearly inverse linear
Tan Q et al. 2020 [50]	Risk of maternal depression	10 studies	90–110 nmol/L (from paper)	*P*_nonlinearity_ = 0.001
Zhang D et al. 2020 [51]	Hypertension	10 studies	Decreasing risk from 75 nmol/L up to lowest risk at 130 nmol/L (from paper)	Restricted cubic splines, L-shaped, *P*_nonlinearity_ = 0.04
Hou Y et al. 2021 [52]	Type 1 diabetes mellitus	10 studies	103–113 nmol/L (from paper)	U-shaped association, inverse nonlinear association (*p* < 0.001)
Jani R et al. 2021 [53]	Fatal CVD events	28 studies	Approx. 30 ng/mL (75 nmol/L) (estimate)	Nonlinear association (*P*_nonlinearity_ < 0.001)
	Non-fatal CVD events	10 studies	Approx. 65 ng/mL (162.5 nmol/L) (rough estimate, end of line)	Linear association
	Combined CVD incidence events	31 studies	Approx. 30 ng/mL (75 nmol/L) (estimate)	Combined CVD events (*P*_nonlinearity_ = 0.001)
Hajhashemy Z et al. 2021 [54]	Abdominal obesity	8 studies	Approx. 85 nmol/L (estimate)	U-shaped, *P*_nonlinearity_ = 0.86
Mohammadi S et al. 2022 [55]	Type 2 diabetes mellitus	19 studies	Approx. 15 ng/mL (37.5 nmol/L, estimate)	U-shaped (*P*_nonlinearity_ = 0.68)
	Type 2 diabetes mellitus + prediabetes	4 studies	Approx. 35 ng/mL (87.5 nmol/L, nearly linear, end of curve, estimate)	*P*_nonlinearity_ < 0.001
Lee K, Kim J 2021 [56]	Metabolic syndrome	23 studies	150 nmol/L (from paper)	Weighted linear dose–response regression model (*P*_nonlinearity_ = 0.10)
Mokhtari E et al. 2022 [57]	Hypertension	10 studies	Around 75 nmol/L (estimate)	Nonlinear association (*P* nonlinearity < 0.001), roughly U-shaped association
Bahadorpour S et al. 2022 [58]	Hypertriglyceridemia	20 studies	Shape of sinusoidal curve; approx. 55 ng/mL (137.5 nmol/L, end of curve, estimate; first nadir at approx. 15 ng/mL)	*P*_nonlinearity_ < 0.001
Guo LL et al. 2022 [59]	Colorectal cancer precursor incidence	7 studies	Approx. 40 ng/mL (100 nmol/L, almost linear, end of line, estimate)	Significant negative dose–response relationship with circulating 25(OH)D (*P*_nonlinearity_ = 0.39) level
Jayedi A et al. 2023 [60]	All-cause mortality in patients with diabetes or prediabetes	11 cohort studies (10 publications)	Around 60 nmol/L (from paper)	J-shaped (*P*_nonlinearity_ < 0.001, *P*_dose–response_ < 0.001)
	Cardiovascular mortality in patients with diabetes or prediabetes	6 cohort studies	Around 60 nmol/L (from paper)	U-shaped (*P*_nonlinearity_ < 0.001, *P*_dose–response_ < 0.001)
Rouhani P et al. 2023 [61]	Preeclampsia	13 publications (nonlinear dose–response analysis)	30 ng/mL (75 nmol/L, from paper)	U-shaped (*P*_nonlinearity_ < 0.001)
Vergatti A et al. 2023 [62]	Recurrent stroke	3 prospective studies	28.1 ng/mL (70.25 nmol/L, from paper)	Nonlinear association (*P*_nonlinearity_ < 0.0001)

* Conversion factor from ng/mL to nmol/L = 2.5; “from paper” relates to data mentioned in publications; “estimate” is approximate value from visual inspection.

## Data Availability

No new data were created or analyzed in this study.
